# Development and validation of a predictive model for complications in robotic thyroid surgery

**DOI:** 10.1007/s13304-026-02776-7

**Published:** 2026-08-12

**Authors:** Yuhan Zhang, Shuai Xue, Jie Luo, Tao Luo, Xiaolin Yue, Bin Luo, Guojun Xue, Fang Chen, Wenxin Su, Peigen Liu

**Affiliations:** 1 Department of General Surgery, Panzhihua Central Hospital, Panzhihua, 617000 Sichuan China; 2 Department of Thyroid Surgery, General Surgery Center, The 1st Hospital of Jilin University, Changchun, China

**Keywords:** Thyroid cancer, Robotic surgery, Surgery-related complications, Predictive model, Learning curve

## Abstract

Robotic thyroid surgery (RTS) has gained popularity due to its minimal invasiveness and improved cosmetic outcomes. However, complications like hypoparathyroidism, bleeding, and infection can occur before surgeons’ learning curve completion. The purpose of present research is to develop and validate a predictive model for complications in RTS prior to their learning curve completion. We retrospectively analyzed data from 236 cases accepted RTS at our institution from Jan 2020 to Dec 2022. The data of included cases were classified into training set (n = 165) and validation set (n = 71). Data on clinical characteristics, surgical details, and postoperative outcomes were collected. Several regression analysis models were applied to identify independent factors of complications. A nomogram was constructed and validated using the training and validation sets. The overall complication rate was 29.24% (69/236 patients). Multivariate analysis identified BMI (odds ratio [OR] = 3.52, 95% Confidence Interval [CI]: 1.179–11.05), Hashimoto’s thyroiditis (OR = 1.69, 95% CI: 1.44–2.05) and tumor size (OR = 2.37, 95% CI: 1.10–5.18) as independent predictors of complications. Based on these predictors, nomogram was constructed. The C-index was 0.8574 (95% CI: 0.762–0.9529) in validation set, indicating good predictive accuracy. This study proposed and validated a nomogram model to predict complications in RTS prior to their learning curve completion. This model can assist clinicians in preoperative counseling and decision-making to minimize surgical risks.

**Supplementary Information:**

The online version contains supplementary material available at https://doi.org/10.1007/s13304-026-02776-7.

## Introduction

Thyroid cancer has shown a rapid increase in global incidence [1]. This upward trend is primarily attributed to the rising detection rates of papillary thyroid carcinoma (PTC), with rates of about 80–85% of all thyroid cancer cases [2]. Recent advances in diagnostic modalities, particularly the widespread adoption of ultrasound and FNA biopsies, have significantly enhanced early detection capabilities. Although surgical intervention remains the cornerstone of PTC management, evolving patient expectations have shifted the therapeutic paradigm.

Given the favorable prognosis of PTC, there is a growing emphasis on maintaining quality of life post-treatment. Conventional open thyroidectomy, while effective, presents significant cosmetic concerns due to prominent neck scarring and functional limitations from anterior cervical tissue damage [3]. Common postoperative complaints include persistent neck numbness, swallowing discomfort, and foreign-body sensations, all of which substantially impact patient satisfaction and quality of life [4].In addition, the traditional open thyroidectomy uses a circular collar-like incision, which may leave permanent scars on the neck of patients with a scar-prone constitution. This affects the mental health of patients, and we found this issue is particularly prominent among young patient groups [5, 6]. These challenges have driven the development of endoscopic technology. Endoscopy can minimize incisions and conceal them in hidden parts of the body through approaches such as transaxillary, transareolar, and transoral access, successfully achieving a scar-free neck [7]. However, endoscopy also has certain limitations, including the lack of a three-dimensional stereoscopic field of view, restricted freedom of movement of surgical instruments, the “chopstick effect”, and the need for surgeons to adapt to non-ergonomic operating postures. These factors collectively result in a relatively long learning curve and potential technical risks. These limitations have driven the development of RTS, which offers distinct advantages in terms of minimally invasive access and superior cosmetic outcomes. However, this innovative technique carries inherent risks, including potential complications like RLN injury, transient or permanent hypoparathyroidism, hemorrhage, and surgical site infections [8].

Current evidence from high-volume centers suggests that robot-assisted thyroidectomy demonstrates safety profiles comparable to conventional surgery when performed by experienced surgeons, with potentially enhanced postoperative quality of life metrics [9–11]. However, the learning curve for this technically demanding procedure remains substantial. Learning curve analyses indicated that surgeons require approximately 50 cases to achieve procedural competence, with complication rates stabilizing only after this threshold [5, 11, 12]. For novice practitioners, careful patient selection during the initial learning phase is crucial for minimizing adverse outcomes. Despite these recognized challenges, current clinical practice lacks evidence-based criteria for identifying high-risk patients predisposed to surgical complications.

To fill this key knowledge gap, we conducted a retrospective analysis of robotic thyroid procedures performed by multiple surgeons in our department prior to their learning curve completion. Through a comprehensive multivariate analysis, we aimed to identify preoperative predictors of surgical complications and develop a validated predictive model. The establishment of such a risk-stratification tool could significantly enhance preoperative decision-making through improved patient counseling and individualized surgical planning. This study is the first systematic investigation into risk factor identification for complications specific to the robotic thyroidectomy learning curve.

## Materials and methods

### Preliminary selection of potential risk factors

**Fig. 1:**
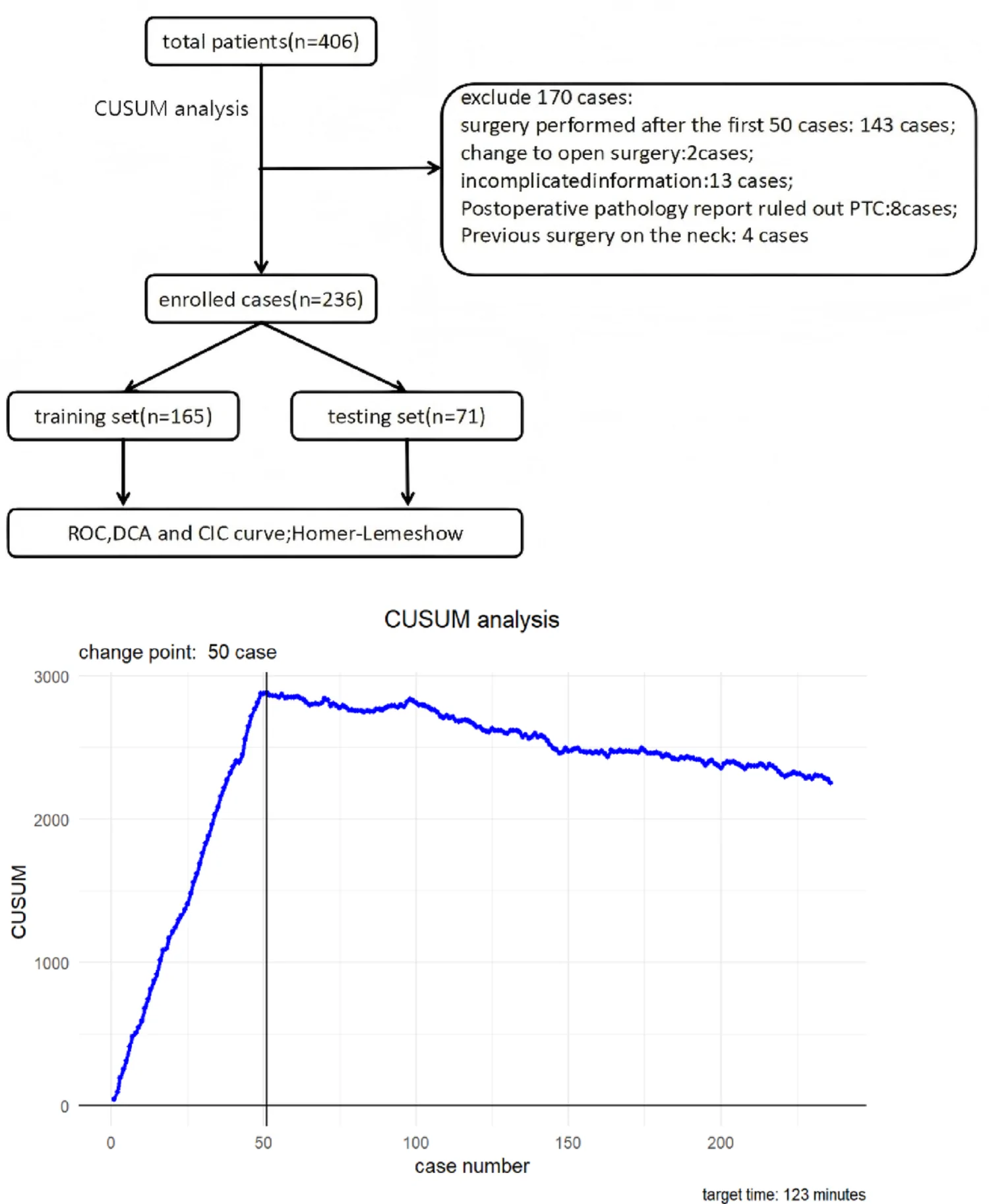
**A** The workflow diagram of this study. ROC, receiver operating characteristic curve; DCA, decision curve analysis; CIC: clinical impact curve. **B** The CUSUM analysis of this study. The purpose of the step is identifying change point of Learning Curve

We retrospectively reviewed 406 patients who underwent RTS at our institution between Jan 2020 and Dec 2022. We completed the CUSUM analysis based on R studio. The study was approved and informed consent was obtained. The inclusion criteria: 1. pathological diagnosis of PTC; 2. Underwent either hemithyroidectomy with central lymph node dissection (CLND) using a robot-assisted approach; 3. The cases are among the first 50 of RTS performed by a surgeon; 4. Complete data; 5. Age above 18 years; 6. No history of neck surgery. According to above criteria, 236 cases were included, with 170 excluded (Fig. 1A). The included cases were divided into control and complication groups according to postoperative complications.

### Surgery and variable assessment

An initial screening was constructed for potential risk factors associated with complications. These potential risk factors included features like age, sex, body mass index (BMI), smoking and alcohol consumption, presence of multifocality, Hashimoto’s thyroiditis (HT), hyperthyroidism, subacute thyroiditis (SAT), and extrathyroidal extension (ETE). tumor size, total thyroidectomy, and tumor location were also considered. Location means the largest tumor’s location in the gland.

### Surgery and variable assessment

Before operation, two physicians use ultrasound equipment to perform thyroid and lymph node ultrasound scans. Under condition of tumor diameter ≥ 0.5 cm, and ultrasound features are suspicious for malignancy, it is necessary to undergo fine needle aspiration (FNA) for further confirmation. The collected samples were reviewed by 3 pathologists, and doubtful cases were discussed until a consensus was reached. The choice of surgical procedure was made based on cases’s condition; hemithyroidectomy with unilateral CLND for patients with unilateral nodules. Robotic bilateral axillo-breast approach (BABA) thyroidectomy involves the creation of four minor incisions: two in the circumareolar area of the breast and two in the axillary region. The surgical field extended superiorly to the cartilage and laterally to the medial edge of the sternocleidomastoid muscle. To sustain the operative space, CO2 insufflation was applied at condition of 5–6 mmHg. The robotic endoscope was inserted via the right breast incision, whereas the both axillary incisions served as access points for the robotic surgical tools. Neuromonitoring was routinely performed interoperating. Using the probe to gently explore areas such as the thyroid lobe and the tracheoesophageal groove, the recurrent laryngeal nerve (RLN) course is identified by the characteristic electromyographic tone and on-screen waveform. Before and after thyroid resection, the vagus nerve and the distal segment of the RLN are stimulated separately; the presence or absence of the signal is used to assess the functional integrity of the nerve.

### Data preprocessing

The dataset was divided into training and testing set with ratio of 7:3, the two set was respectively used to train and validate the model, so as to meet the requirement. Baseline clinicopathological characteristics were compared in Table 1. RLN injury was defined as an intraoperative decrease of more than 50% in RLN monitoring signal coupled with postoperative laryngoscopy evidence of vocal cord immobility. Hypoparathyroidism was characterized by postoperative levels of parathyroid hormone that fall below the normal reference range. Complications that resolve within six months post-surgery were classified as temporary. Cases that persisted beyond this period were considered permanent. In the context of RTS, the conversion to open surgery refers to the intraoperative necessity to switch from a robotic approach to a conventional open surgery due to factors such as bleeding, which impede the continuation of the robotic procedure.

**Table 1:** Baseline characteristics of enrolled patients

Characters	Total (n = 236)	Train (n = 165)	Test (n = 71)	*p*-value
Age, median [IQR]	43.48 [32.25,52]	43.86 [37,53]	41.85 [34,51]	0.137
Sex, n (%)				0.057
Male	200 (84.75)	144 (87.27)	56 (78.87)	
Female	36 (15.25)	21 (12.73)	15 (21.13)	
BMI, median [IQR]	24.56 [21.88,27.57]	24.86 [21.88,27.89]	24.30 [21.76,26.87]	0.268
HT, n (%)				0.056
No	166 (70.34)	126 (76.36)	40 (56.24)	
Yes	70 (29.66)	39 (23.64)	31 (43.66)	
Hyperthyroidism, n (%)				0.66
No	213 (90.25)	148 (62.71)	65 (91.55)	
Yes	23 (9.75)	17 (7.20)	6 (8.45)	
SAT, n (%)				0.485
No	226 (95.76)	159 (67.37)	67 (94.37)	
Yes	10 (4.24)	6 (3.64)	4 (5.63)	
Tumor_size,median [IQR]	0.96 [0.70,1.20]	1.01 [0.70,1.25]	0.98 [0.65,1.10]	0.812
TT				0.053
No	164 (73.73)	128 (77.58)	36 (50.70)	
Yes	72 (30.51)	37 (22.42)	35 (49.30)	
Multifocus, n (%)				0.782
1	170 (72.03)	120 (72.73)	50 (70.42)	
2	38 (16.10)	25 (15.15)	13 (18.31)	
≥ 3	28 (11.86)	20 (12.12)	8 (11.27)	
ETE, n (%)				0.822
No	127 (53.81)	88 (53.33)	39 (54.93)	
Yes	109 (46.19)	77 (46.67)	32 (19.39)	
Location, n (%)				0.053
Upper	72 (30.51)	51 (30.91)	21 (29.58)	
Middle	119 (50.42)	83 (30.30)	36 (50.70)	
Lower	39 (16.53)	27 (16.36)	12 (16.90)	
Isthmus	6 (2.54)	4 (2.42)	2 (2.82)	
Smoke, n (%)				0.569
No	204 (86.44)	144 (87.27)	60 (84.51)	
Yes	32 (13.56)	21 (12.73)	11 (15.49)	
Alcohol, n (%)				0.934
No	182 (77.12)	127 (76.97)	55 (77.46)	
Yes	54 (22.88)	38 (23.03)	16 (22.53)	
Complications, n (%)				0.389
No	167 (70.76)	114 (69.09)	53 (74.65)	
Yes	69 (29.24)	51 (30.91)	18 (25.35)	

*IQR* interquartile range, *BMI* body mass index, *HT* hashimoto’s thyroiditis, *SAT* subacute thyroiditis, *TT* total thyroidectomy, *ETE* extrothyroidal extension

### Statistical analysis

The baseline features of the control and complication groups were compared using R Studio. The variable was univariate analyzed to judge that if there exist statistically differences. The statistically difference judgement criteria was set as *p* < 0.05. Chi-square tests and Mann–Whitney U tests were respectively applied for categorical and continuous data.

### Model building and validation

A stepwise backward binary regression model was constructed based on R Studio, and the model was used to identify risk factors for the complication group. The variables were selected according to their influence to the prediction of complications. The nomogram was drawn based on the variables selected by the above constructed model. This nomogram can be used to analysis the predictive value of the model. To assess the performance of model, we applied receiver operating characteristic (ROC) curves and calculated the index of Area Under Curve (AUC). The goodness-of-fit test to evaluate the model calibration, with a *p*-value above 0.05 indicating high calibration. The clinical decision curve was used to judge the clinical utility, so as to judge the clinical value and prediction effect of the model. The Brier score was determined to overall assess the model’s effect. This score is positively associated with the accuracy of the model.

## Results

### Patient characteristics

In present research, 236 cases met the inclusion criteria. 69 patients had operative complications, while the remaining 167 were classified into the control group (no complications). The result of CUSUM analysis was showed in Fig. 1B. It was demonstrated that change point is the 50th case. Complications include infection (0.42%), bleeding (0.85%), temporary hypoparathyroidism (9.74%), temporary RLN injury (12.28), permanent hypoparathyroidism (2.54%), permanent RLN injury (3.81%) (Table S1). The cases were divided into training and testing sets. No significant differences were observed in the basic characteristics between the two datasets (Table 1). In the training set, baseline comparisons of features indicating obvious differences in the complication positive and negative groups in terms of BMI, HT, tumor diameter, and TT. These results were existed in Table 2.

**Table 2:** Univariable analysis for risk factors of complications in the training set

Characters	Complication negative (n = 114)	Complication positive (n = 51)	*P*-value
Age, median [IQR]	44.47 [36.00,55.00]	42.49 [38.00,48.00]	0.124
Sex, n (%)			0.797
Male	14 (12.28)	7 (13.72)	
Female	100 (87.72)	44 (86.27)	
BMI	23.28 [20.80,24.98]	28.40 [27.01,29.99]	0.001
HT, n (%)			0.002
No	95 (83.33)	31 (60.78)	
Yes	19 (16.67)	20 (39.22)	
Hyperthyroidism,n(%)			0.128
No	105 (92.11)	43 (84.31)	
Yes	9 (7.89)	8 (15.69)	
SAT, n (%)			1.000
No	110 (96.49)	49 (96.08)	
Yes	4 (3.51)	2 (3.92)	
tumor_size	0.96 [0.70,1.10]	1.13 [0.70,1.50]	0.005
TT, n (%)			0.002
No	96 (84.21)	32 (62.75)	
Yes	18 (15.79)	19 (37.25)	
Multifocus, n (%)			0.094
1	88 (77.19)	32 (62.75)	
2	16 (14.03)	9 (17.65)	
≥ 3	10 (8.77)	10 (19.61)	
ETE, n (%)			0.787
No	60 (52.63)	28 (39.44)	
Yes	54 (47.37)	23 (60.56)	
Location, n (%)			0.690
Upper	37 (32.46)	14 (27.45)	
Middle	58 (50.88)	25 (49.02)	
Lower	17 (14.91)	10 (19.61)	
Isthmus	2 (1.75)	2 (3.92)	
Smoke, n (%)			0.451
No	98 (85.96)	46 (90.20)	
Yes	16 (14.04)	5 (9.80)	
Alcohol, n (%)			0.485
No	86 (75.44)	41 (80.39)	
Yes	28 (24.56)	10 (19.61)	

*IQR* interquartile range, *BMI* body mass index, *HT* hashimoto’s thyroiditis, *SAT* subacute thyroiditis, *TT* total thyroidectomy, *ETE* extrothyroidal extension

### Development of the model

**Fig. 2:**
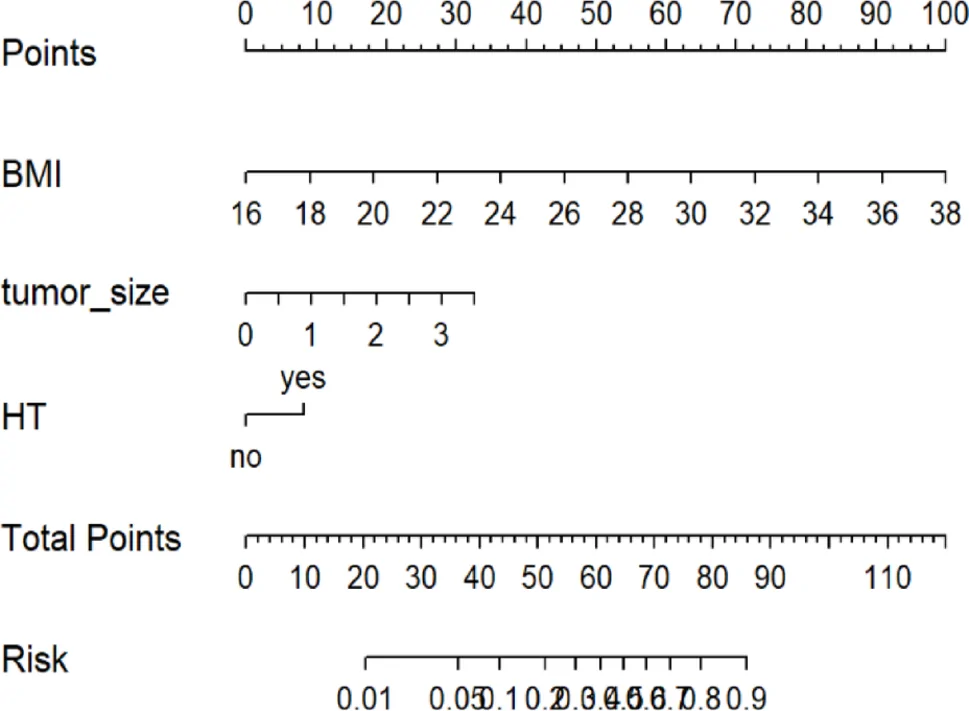
Graphic nomogram based on a multivariable logistic regression model for prediction of patients underwent RTS with complication. RTS: robotic thyroid surgery

The R Studio was applied to establish multivariate regression analysis on all variables to identify risk factors related to complications. The results yielded the following factors with their relevant odds ratios (OR) and 95% CI: BMI (3.52 [1.179–11.05]), HT (1.69 [1.44, 2.05]) and tumor size (2.37 [1.10–5.18]) (Table 3). A nomogram was constructed based on these factors (Fig. 2). Among them, BMI exhibited the highest OR, indicating more influence on outcome prediction.

**Table 3:** Multivariable logistic regression analysis for predictive factors of complications during robotic thyroid surgery

	OR [95%CI]	*P* value
BMI	3.52 [1.179–11.05]	0.000
HT: yes	1.69 [1.44–2.05]	0.027
Tumor size	2.37 [1.10—5.18]	0.026

*BMI* body mass index, *HT* hashimoto’s thyroiditis, *OR* odds ratio, *CI* confidence interval

### Validation of the model

**Fig. 3:**
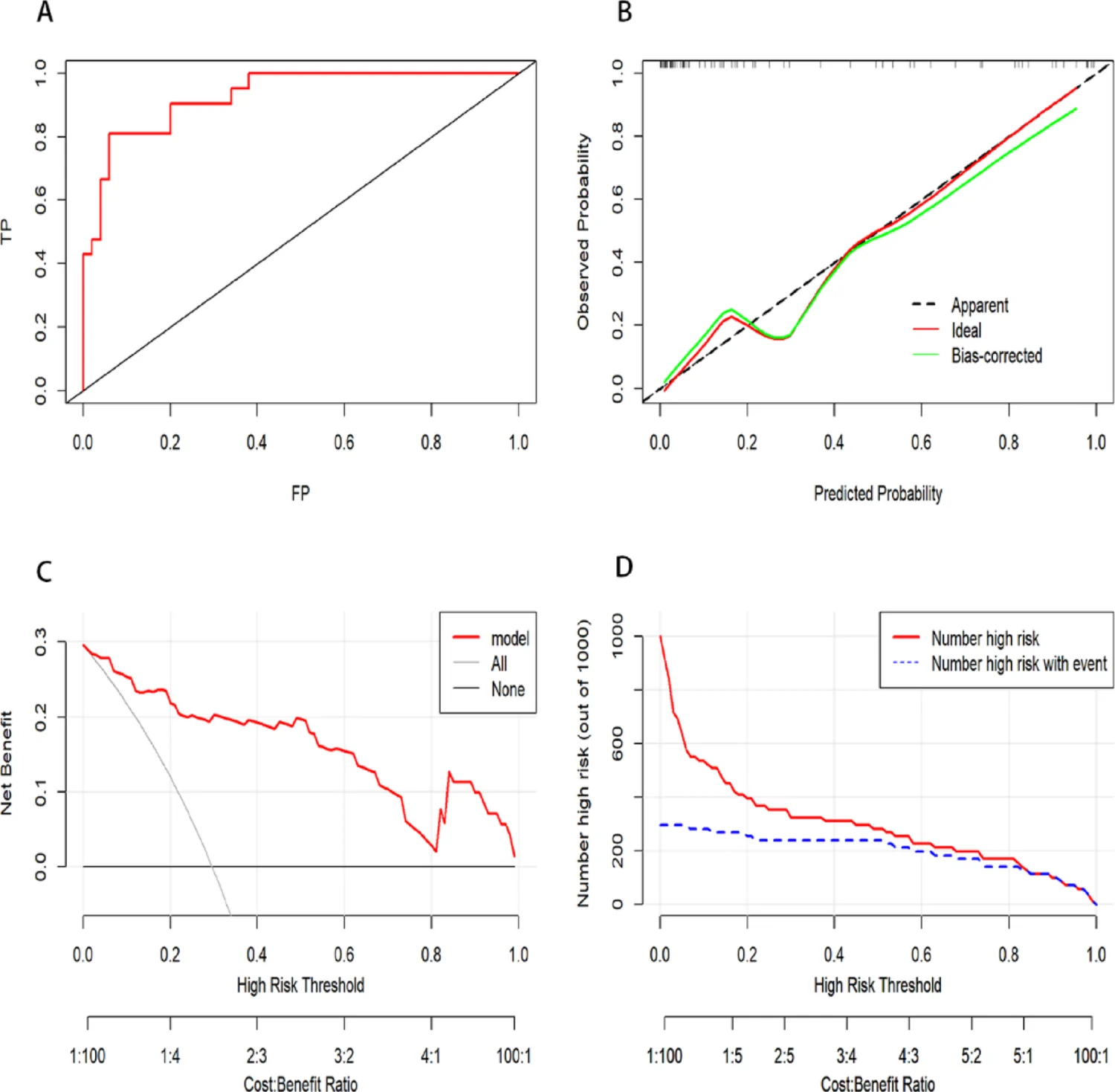
**A** The ROC analysis with AUC for training set. **B** Discrimination plot for training set. **C** DCA revealed that patients would benefit more if this model was used to predict the risk of complication when the threshold probability is between > 1% and < 80% in the training set. **D** The CIC of the training set revealed that the patients categorized as complication positive by the nomogram were more likely to be truly positive when the threshold probability < 80%. ROC, receiver operating characteristic curve; AUC, area under curve; DCA, decision curve analysis; CIC: clinical impact curve

To prove the accuracy of the above constructed model, the R Studio tool was applied to draw an ROC curve (Fig. 3A). The AUC was determined to be 0.8913, which suggest a fact that the model’s precision in predicting complicated cases. According to the calibration curve, it can be inferred that there exist high alignment between the model’s predicted result and the actual value. In the goodness-of-fit test, the *p*-value was 0.1627. According to the above results, the model’s predicting performance was proved (Fig. 3B).

The decision curve analysis (DCA) result indicats that the model’s net benefit was obviously higher than that of the two extreme curves, which reflects that larger cut-off values could be chosen to enhance the performance of model (Fig. 3C). In the clinical impact curve (CIC) the false positive rate was obviously decreased at threshold of 0.2 (Fig. 3D). The mean absolute error was 0.002, which suggests that higher threshold is safe, and can ensure the safety of the model.

The model was further validated on the testing set, and the Supplementary Material exhibits the obtained results (Fig. S1). The AUC was 0.8574 (95% CI: 0.762–0.9529), and the calibration curve indicate that the model has good fiteffect. The *p*-value for the goodness-of-fit test was 0.757. Similar to the training set, both the DCA and CIC curves have wide threshold ranges, thereby the calibration performance of the model was further proved.

## Discussion

This research analyzed clinical data from 236 cases who accepted RTS. The aim was to identify key risk factors influencing surgical complications and to establish a predictive model. The results indicated that BMI, HT, and tumor size are independent predictors of complications in RT. Based on these factors, we developed a predictive model and validated its accuracy and clinical utility through ROC curves, calibration curves, DCA, and CIC. The novelty of present research lies in its systematic exploration of risk factors for complications during the learning curve period of RTS and the construction of a tool for preoperative risk assessment. This model not only helps optimize patient selection, but also provides decision-making support for surgeons during their technical learning process, thereby reducing complication rates and improving surgical safety.

**Fig. 4:**
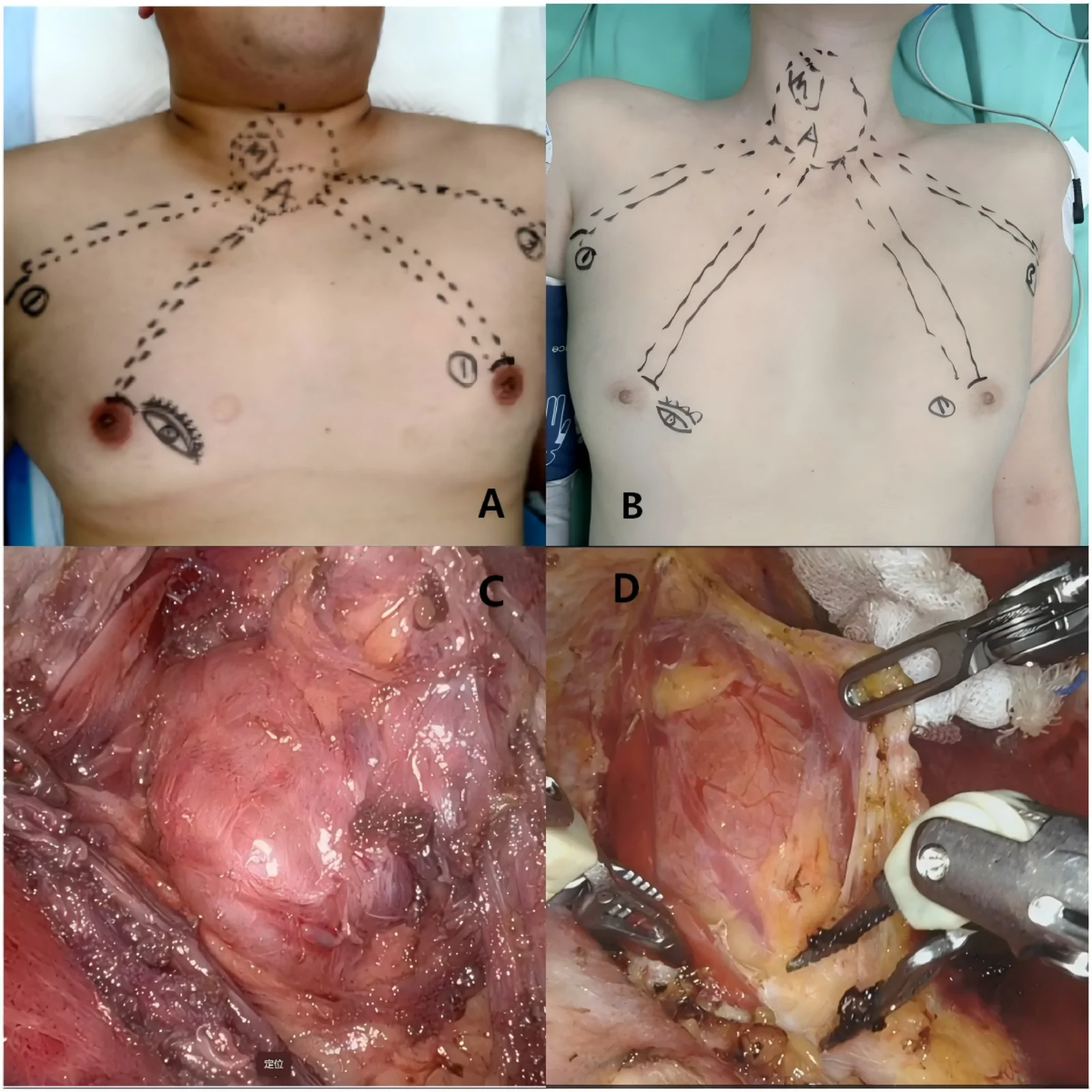
**A** A surgical area picture of obese patient. **B** A surgical area picture of thin patient. A and B show the intraoperative differences between patients of two body types. **C** Intraoperative pictures for thyroid with HT. **D** Intraoperative pictures for thyroid with HT. C and D show different states of glands

We found that BMI was the strongest predictor of complications after RTS. Figure 4A and B show the intraoperative differences between patients of two body types. According to other relevant research, BMI did not correlate with complications after surgery, such as RLN palsy, bleeding, chyle leakage [13]. However, the robotic surgeries were performed by experienced surgeons and the complication rate was relatively low (13%) [13]. Patients with a high BMI may face higher surgical risks [14], primarily for the following reasons. First, the anatomical structure of the neck in obese patients is more complex [15]; increased adipose tissue may limit the surgical field of view, thereby increasing the difficulty of the procedure. Second, patients with a high BMI often have thicker neck soft tissues, which may affect the operational space and flexibility of robotic instruments, leading to prolonged surgical time and increased procedural complexity [16, 17]. Additionally, obese patients tend to have poorer wound-healing capabilities and a higher risk of infection, which may also contribute to increased complication rates. Therefore, more detailed preoperative assessments should be conducted for patients with high BMI, and more cautious surgical strategies should be adopted to reduce the risk of complications. Moreover, it is very necessary to collect more samples, so as to improve the performance of the model.

HT was another significant independent risk factor identified in this study. A comparison of thyroid tissue from patients with and without HT is presented in Fig. 4C and D. Thyroid tissue in patients with HT is often accompanied by chronic inflammation and fibrosis, which may complicate tissue dissection during surgery. Moreover, the thyroid volume in HT patients may be enlarged, with more severe adhesions to surrounding tissues, further increasing procedural difficulty. Studies have also shown that patients with HT have a higher incidence of postoperative hypocalcemia, which may be related to impaired parathyroid function [18]. Therefore, for patients with HT, preoperative assessment of thyroid and parathyroid functions should be thorough. Special attention needs to focus on protecting the parathyroid glands and RLN in operation process to reduce postoperative complication.

Tumor size was the third independent risk factor identified in this study. Larger tumors may exert pressure on or infiltrate surrounding tissues (such as the RLN, parathyroid glands, and trachea) [19], increasing the difficulty and risk of surgical dissection. Many studies have shown that RTS can treat larger thyroid cancers or nodules without increasing complications when performed by experienced surgeons [20, 21]. However, patients with larger tumors often require extensive surgical resection, which may prolong the surgical time and increase the risk of intraoperative bleeding during the learning curve, and is harmful for the patients. Therefore, for patients with larger tumors, preoperative imaging should thoroughly assess the extent of the tumor and its association with nearby structures.

Although this study provides valuable clinical insights, it has some limitations. First, as this was a single-center study with small sample size, there may be selection bias. Second, this study only included patients who underwent RTS and did not compare them with those who underwent traditional open surgery, making it impossible to directly evaluate the superiority of robotic surgery. Although we controlled for some confounding factors using multivariate analysis, unmeasured confounders may still exist. It was hard to perform statistical analysis independently since there were only 1–2 cases of each consequence, such as bleeding and infection. Future studies should expand the sample size, incorporate multicenter data, and adopt prospective designs to further validate the findings of this study. Furthermore, integrating artificial intelligence technology and imaging data may further enhance the accuracy and clinical applicability of the predictive model to promote the further application of it.

In conclusion, this study identified BMI, HT, and tumor size as independent risk factors for complications in RTS before surgeons’ learning curve completion. An effective predictive model was also constructed, providing an important decision-support tool in clinical practice. Future research should further refine this model and explore its applicability in broader populations.

## Supplementary Information

Below is the link to the electronic supplementary material.

Supplementary Material 1

Supplementary Material 2

Supplementary Material 3

## Data Availability

The datasets generated and analyzed during the current study are available from the corresponding author upon reasonable request.
