# Supplementary material for: Development and validation of a predictive model for complications in robotic thyroid surgery
Source: Updates Surg. 2026 Aug 12;78(7):2893–901. doi: 10.1007/s13304-026-02776-7 (PMC13751752; doi:10.1007/s13304-026-02776-7)
Supplement: Supplementary file 1 [file 13304_2026_2776_MOESM2_ESM.docx]

| Complication | n | Proportion (%) |
| --- | --- | --- |
| infection | 1 | 1.45% |
| bleeding | 2 | 2.89% |
| Temporary hypoparathyroidism | 23 | 33.33% |
| Temporary recurrent laryngeal nerve injury | 28 | 40.6% |
| Permanent hypoparathyroidism | 6 | 8.69% |
| Permanent recurrent laryngeal nerve injury | 9 | 13.04% |

Table S1

The classification of complications
